# Intraoperative variability of corneal epithelium thickness in photorefractive keratectomy

**DOI:** 10.1007/s10792-024-03154-1

**Published:** 2024-06-25

**Authors:** Dana Barequet, Eliya Levinger, Amir Rosenblatt, Samuel Levinger, Irina S. Barequet

**Affiliations:** 1 Division of Ophthalmology, Tel Aviv Medical Center, Tel Aviv University, 6 Weizmann Street, 64239 Tel Aviv, Israel; 2 Enaim Refractive Surgery Center, Tel Aviv, Israel; 3 Goldschleger Eye Institute, Sheba Medical Center, Tel Hashomer, Israel; 4 Sackler Faculty of Medicine, Tel Aviv University, Tel Aviv, Israel

**Keywords:** Corneal epithelium, Epithelium thickness, Photorefractive keratectomy, Optical coherence pachymetry

## Abstract

**Purpose:**

To evaluate the intraoperative central corneal epithelial thickness (ET) as measured by optical coherence pachymetry (OCP) in myopic eyes undergoing alcohol-assisted photorefractive keratectomy (PRK).

**Methods:**

A retrospective review of patients who underwent alcohol-assisted PRK was performed. Data were abstracted on age, gender, contact lens (CL) wear, preoperative refractive errors, keratometry, topographic and ultrasonic pachymetry, and intraoperative OCP measurements before and after epithelium removal. The central ET was calculated by subtracting OCP measurement after epithelium removal from the OCP measurement prior to epithelium removal.

**Results:**

The study comprised of 162 consecutive eyes from 81 patients. Mean age was 26.73 ± 6.47 years, 50.6% were males. CL was used in 92 eyes (56.8%). The mean sphere and spherical equivalent were −3.60 ± 1.84 D and −3.26 ± 1.85D, respectively. The mean intraoperative ET was 58.22 ± 17.53 µm (range, 15–121µm). Fifty-five percent of the eyes had an ET measurement above or below the range of 40-60µm. ET was significantly higher in the second operated eye compared to the first operated eye (*p* = 0.006), and an association was found to CL-wear (*p* = 0.03). There was no significant difference in thickness between genders (*p* = 0.62), and no correlation to patient age (*p* = 0.45, r_p_ = 0.06), refractive errors (*p* > 0.30,r_p=_−0.07–0.08), nor keratometry(*p* > 0.80, r_p=_−0.01− (−0.02)).

**Conclusion:**

The intraoperative assessment of ET in alcohol-assisted PRK showed a high variability of the central corneal epithelium, with a significant difference between the first and second operated eyes. This difference may have implications when the epithelium is not included in the surgical planning in surface ablation.

## Introduction

The corneal epithelium has been previously recognized to have a distinct profile [1]. The epithelium plays an important role in the corneal power and accounts for an average of 1.03 diopters [2]; it has an accepted average thickness of approximately 50–60 µm [1, 3]. Several factors have been shown to influence the central epithelial thickness profile, and while controversial in the literature, they include age [4, 5], gender [4, 6], ethnics and refractive errors [6, 7]. Pathological conditions, such as contact lens (CL) wearing [8], dry eye [9, 10], and keratoconus [11], have been also shown to cause alterations of ET.

Numerous novel imaging modalities, such as high-frequency scanning ultrasound biomicroscopy [3], confocal microscopy [12, 13], and optical coherence tomography (OCT) [10, 14, 15], have been developed in the last decade and have increased the attention to the importance of epithelial thickness mapping. These devices map the epithelium by differentiating it from the underlying corneal layers and are utilized in the diagnosis and follow-up of various corneal disorders as well as in the preoperative evaluation of refractive-surgery candidates. The optical coherence pachymetry system (OCP, Heidelberg Engineering, Lübeck, Germany) is integrated in the SCHWIND Amaris excimer laser, providing non-contact continuous measurements of the central corneal thickness during refractive procedures. It allows for intraoperative monitoring of the central corneal thickness throughout the entire surgery. The OCP has been shown to have high reproducibility of intraoperative corneal changes, such as the flap and the residual stromal thickness [16].

During photorefractive keratectomy (PRK), the treatment is performed on the Bowman’s layer and corneal stroma, while the epithelium is removed either manually or alcohol-assisted. Therefore, the epithelium does not play a role in the refractive outcome. Recently, transepithelial PRK (t-PRK) has been gaining popularity due to its being a single-step surgery composed of a fixed predefined epithelial ablation of 55 μm at the center of the cornea and 65 μm at the periphery, followed by stromal ablation in a continuous profile [17]. However, individual variations along with intraoperative changes of ET may lead to incomplete removal or overtreatment of the epithelium (when the epithelium is removed in a fixed preset depth) that may affect the refractive outcome. Intraoperative assessment of the epithelial thickness may provide information on the real-life changes of the epithelium doing the procedure.

To the best of our knowledge, the intraoperative central corneal epithelium measurement has not been previously reported. The purpose of our study was to evaluate the intraoperative ET in alcohol-assisted PRK.

## Materials and methods

### Patient and study design

This study was a retrospective evaluation of eyes that underwent bilateral simultaneous alcohol-assisted myopic PRK between October 2018 through May 2019. Indications for the PRK included eyes with corneal thickness greater than 480 µm, no contraindications for laser vision correction, and refractive errors indicated clinically being correctable with an aspheric PRK profile. We included patients with complete pre- and intraoperative data as described below and excluded from the analysis patients who underwent unilateral PRK or missing complete data on both eyes. Patients received a detailed written informed consent form prior to surgery. The study was approved by the Institutional Review Board at the Sheba Medical Center and complied with the Declaration of Helsinki.

### Preoperative examination

A detailed ophthalmic and systemic history was obtained. CL wear was discontinued at least 10 days, depending on CL type, prior to the preoperative evaluation and surgery. Preoperative examination included uncorrected distance visual acuity, best corrected distance visual acuity, manifest and cycloplegic refraction, a full ophthalmic examination including slit-lamp examination, intraocular pressure by Goldmann applanation tonometer measurement, corneal epithelium assessment by fluorescein staining, tear breakup time, and dilated fundus examination. In addition, all patients underwent Scheimpflug tomography (Sirius, CSO, Florence, Italy) and central ultrasound pachymetry.

### Surgical technique

All surgeries were performed using the same technique by 2 surgeons (I.S.B. and E.L.), using the SCHWIND Amaris 500E excimer laser platform (SCHWIND eye-tech-solutions GmbH, Kleinostheim, Germany). The OCP platform integrated in the laser system was utilized for the intraoperative central pachymetry measurements. The ablation algorithm was calculated using ORK-CAM software. The target refraction was emmetropia in all eyes.

In the preparation area, one drop of topical 0.4% oxybuprocaine hydrochloride (Localin, Fischer Pharmaceuticals Ltd., Bney Brak, Israel) and moxifloxacin (Vigamox, Alcon Laboratories, Inc., Fort Worth, TX) were instilled in the eyes, and the lid margins were cleaned with a 5% povidone iodine solution. Immediately afterwards, the patient entered the laser room to the supine position of the laser system. A sterile drape was placed at the lid margins and a lid speculum was inserted. The first OCP measurement of the corneal thickness was performed while the tracker was aligned on the eye. Another drop of topical 0.4% oxybuprocaine hydrochloride anesthesia was instilled in the operated eye, and, then, epithelial delamination was achieved with an 8.5-mm well-placed centrally on the cornea and filled with 20% ethanol alcohol for 30 s, followed by absorption with a Merocel sponge. The epithelium was debrided using a blunt spatula and the Bowman layer was exposed. A second OCP measurement was obtained prior to the laser ablation in an identical alignment as the first measurement. The laser ablation was performed, and Mitomycin C 0.02% was applied to the stromal bed for 10 to 30 s depending on the ablation depth. The bed was irrigated with 20 mL of chilled balanced salt solution, and a soft bandage contact lens was placed for four to 6 days. Patients were instructed to use 0.5% moxifloxacin eye drops four times daily for 7 days; 0.5% loteprednol eye drops (Lotemax, Bausch & Lomb, Inc.) four times daily for a month, and then tapered down gradually over the next month; and non-preserved artificial tears as needed.

### Data collection

Data were abstracted for age, gender, contact lens wear, refractive errors, and keratometry (Javal keratometry). Central corneal pachymetry data were obtained as recorded from the ultrasound pachymetry and the tomographic parameter of minimal corneal thickness. Intraoperative data of the OCP measurement were recorded. The central ET was calculated by subtracting OCP measurement after epithelium removal from the OCP measurement prior to epithelium removal.

### Statistical analysis

Data were recorded in Microsoft Excel 2010 and analyzed using SPSS version 25 (SPSS Inc., Chicago, IL, USA).

Continuous variables, such as epithelial thickness, in respect to dichotomic demographic data, were compared between subjects using the independent sample t-test. In cases of paired variables, such as right and left comparison, data were restructured and paired sample t-test was used.

Correlation of continuous variables was examined using the Pearson's correlation.

All tests were 2-tailed, and the threshold for statistical significance was defined as a *p*-value < 0.05.

## Results

The study included 162 eyes of 81 consecutive patients. The mean age of the patients was 26.73 ± 6.47 years (range, 18–45), 50.6% were males. Contact lens wear was reported to be used in 92 eyes (56.8%). The preoperative refractive measurements for all eyes included a mean sphere of −3.76 ± 2.63 D (range, −8.00 to −0.25 D); a mean cylinder of -0.68 ± 0.68 D (range, −5.00 to 0 D); and a mean spherical equivalent of −3.26 ± 1.85 D (range, −7.88 to + 0.375 D). Table 1 demonstrates patients’ baseline characteristics.

**Table 1:** Association between epithelium thickness to demographics and preoperative measurements

	Patients *n* = 81 n = 20	Eyes *n* = 162	*p*	*r*_p_
Age^a^, years (mean ± SD)	26.73 ± 6.47		0.45	0.06
Gender-male, n (%)	41 (50.6)		0.62	
History of contact lens, n (%)		92 (56.8)	0.03	
*Refractive errors, D (mean* ± *SD)*				
Sphere		− 3.60 ± 1.84	0.37	0.07
Cylinder		− 0.68 ± 0.68	0.40	-0.07
Spherical equivalent		− 3.26 ± 1.85	0.30	0.08
*Keratometry, D (mean* ± *SD)*				
K1		43.49 ± 1.64	0.86	-0.01
K2		44.39 ± 1.64	0.83	-0.02
K average		43.94 ± 1.61	0.80	-0.02
*Preoperative pachymetry, µm (mean* ± *SD)*				
US pachymetry		540.88 ± 30.50	0.81	0.02
Topography (Sirius)		542.98 ± 31.15	0.29	0.08
Optical coherence pachymetry		537.22 ± 35.58	0.001	0.33

*US* ultrasound

^a^Age: Age at first examination

Intraoperative corneal thickness measurements were significantly correlated to the ET measurements (*p* < 0.001, r_p_ = 0.33), as well as to the ultrasonic pachymetry and tomography measurements (*p* < 0.001, r_p_ = 0.77 and R_p_ = 0.85, respectively).

**Fig. 1:**
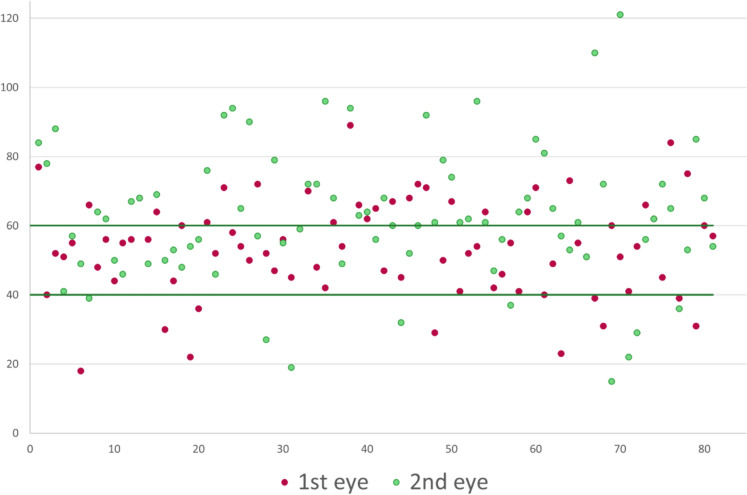
Intraoperative measurements of epithelium thickness (y-axis) for each eye (x-axis)

The mean epithelium thickness was 58.22 ± 17.53 µm, ranging from 15 to 121 µm (Fig. 1). Seventy-three eyes (45.1%) had an ET within the range between 40 and 60 μm, while in the remaining 89 eyes (54.9%) the ET was either below (19 eyes, 11.7%) or above (70 eyes, 43.2%) these values.

There was no significant difference in ET between males and females (p = 0.62). Contact lens users were found to have a significantly thinner epithelium thickness compared to non-contact lens users (55.64 µm compared to 61.61 µm, *p* = 0.03). Epithelium thickness was not correlated to patient age (*p* = 0.45, r_p_ = 0.06), refractive errors (*p* > 0.30, r_p=_−0.07–0.08), nor to keratometry (p > 0.80, r_p=_−0.02 − ) −0.01)). The results are summarized in Table 1.

**Fig. 2:**
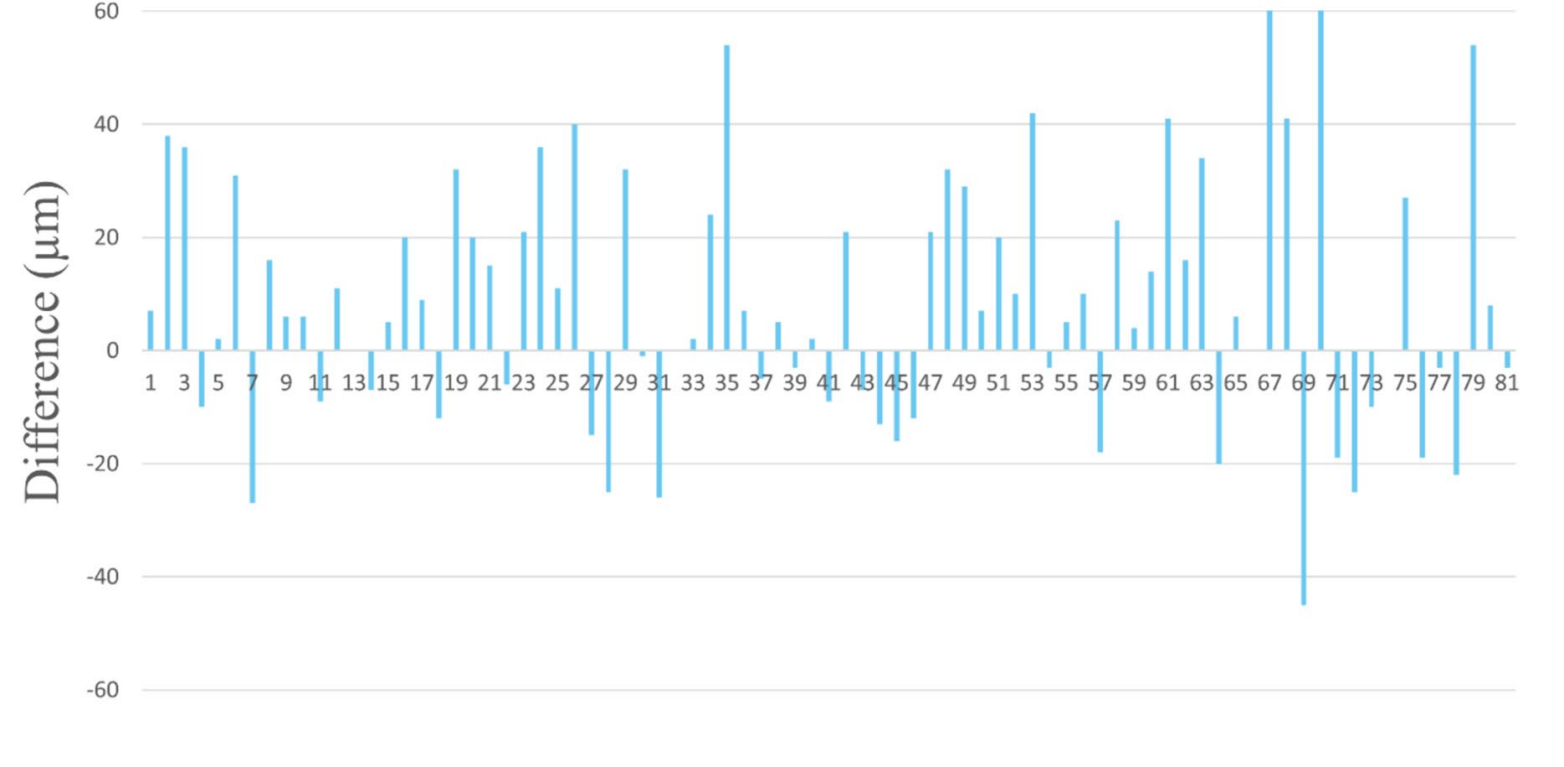
Epithelium thickness difference between second and first operated eye

While comparing the first and second operated eyes’ ET for each patient, we found that the ET in the second operated eye was significantly thicker than the first operated eye (62.57 μm compared to 53.88 μm, p < 0.001). Figure 2 illustrates the difference between the second and first operated eye for each patient, showing an increase in thickness of the second eye for the vast majority of the patients.

## Discussion

To our best knowledge, this is the first study to assess the intraoperative central corneal epithelium thickness during PRK. Utilizing the OCP integrated in the SCWIND Amaris excimer laser platform, we found an overall high variability of the central corneal epithelium. Moreover, the outcomes demonstrated that the second operated eye had significantly thicker central epithelium than the first operated eye.

Several strategies have been reported in the literature for the measurement of epithelium thickness, including VHF digital ultrasound [1, 3], optical pachymetry [7, 18], anterior-segment OCT[4–6, 8–10, 19] and confocal microscopy [20]. The advances in imaging techniques enabled clinical applications of ET mapping. This tool is limited to the preoperative evaluation of refractive surgery candidates. Although preoperative testing may provide data of the individual epithelium profile to diagnose subclinical disorders, these maps, do not necessarily reflect intraoperative changes in the epithelium that may affect the ablation impact.

The OCP is used for continuous monitoring of the corneal thickness intraoperatively. OCP measurements have been previously reported to show a high reproducibility [21–23]. These reports are in agreement with the measurements of the total corneal thickness in our study, in which the initial intraoperative OCP measurement of corneal thickness was significantly correlated to all preoperative corneal thickness measurements, both by US pachymetry and topography.

The calculated mean central ET in our study was 58.22 ± 17.53, similar to previous preoperative assessments that reported a mean ET between 48 ± 5 μm [18] and 59.9 ± 5.9 μm [7]. However, the wide range of the intraoperative ET measurements (between 15 and 121 µm) highlights the intraoperative changes that can occur at the individual level during the operation. This supports the need for caution when referring intraoperatively to preoperative measurements.

In addition, when comparing consecutive eyes for each patient, we found that the corneal epithelium of the second operated eye was significantly thicker than that of the first operated eye (54.8 ± 17.6 µm compared to 64.1 ± 21.1 µm, *p* = 0.006). This is opposed to previous preoperative assessments[3, 14, 23] showing no difference in epithelium thickness between right and left eyes. The ET difference between fellow eyes when measured intraoperatively, may be caused by several factors related to the time gap between the first and second operated eyes. First, there is agap time between the measurement of each eye while the patient lays supine in the laser suite, enabling different exposure rates of the epithelium. The first eye is measured immediately in the beginning of the surgery, while the second eye is assessed after completing the laser operation of the first eye. Second, the anesthetics drops are instilled in both eyes prior to the entrance to the operating room.Several studies reported that the topical anesthetic agents [24–27] can cause a small transient increase in central corneal thickness. Moreover, Mukhopadhyay et al.[25] found a large degree of interindividual variability in the amount of swelling, ranging from a decrease of more than 10 µm to an increase of over 30 µm in individual cases. Weekers et al.[26] revealed an alteration of the Na + /K + endothelium pump on the corneal epithelium in rabbits, resulting in increased osmotic pressure and subsequent increased hydration of the epithelium and stroma. In our study, the high variability of ET and the difference between the first and the second operated eye may be explained by the greater influence of the anesthetic drops on the second operated eye, as more time elapsed between the installation and the surgery.

The limitations of our study are to its retrospective nature, although by collecting data on consecutive eyes we attempted to minimize collection bias. Another limitation is related to the indirect assessment of ET values, calculated by subtracting OCP measurements before and after epithelium removal. However, the measurements were performed at the same setting and eye tracking alignments.

Transepithelial PRK (SCHWIND eye-tech-solutions GmbH, Kleinostheim, Germany) is becoming a popular refractive procedure [27–29]. A single step standard epithelial ablation pattern of 55 μm centrally is performed followed immediately by the stromal ablation. Although many researchers showed the efficacy and safety of t-PRK [27–29], concerns were raised about possible effect of interindividual epithelial thickness profile variability on the refractive outcomes [30, 31]. A study by Jun et al.[32] showed a significant difference in postoperative sphere and spherical equivalent between groups of ET undergoing t-PRK, with a mild hyperopic shift in the group of ET < 50 μm, and a slight myopic shift in the 60 μm or greater ET group. This phenomenon can be explained by less stromal ablation at the same ablation depth setting. Our intraoperative cohort showed that 45.1% of the eyes had an ET within the range between 40 and 60 μm, while 54.9% were below or above these values. The variation of ET found in our study can be similarly applicable to t-PRK and supports the need of a real-life assessment of the ET and addressing the measurements in the excimer laser ablation profile.

## Conclusion

The intraoperative OCP measurements during alcohol-assisted PRK showed a high variability of central corneal epithelium thickness, with the second operated eye being significantly thicker than the first operated eye. The intraoperative measurements may differ from the preoperative evaluation and may have potential implications (under- or overcorrections) on the refractive outcomes of surface keratorefractive surgery when not included in the surgical planning. Additional studies correlating between the preoperative epithelial profile and intraoperative measurements are warranted.
